# Descending serotonergic facilitation mediated by spinal 5-HT3 receptors engages spinal rapamycin-sensitive pathways in the rat

**DOI:** 10.1016/j.neulet.2010.08.024

**Published:** 2010-10-29

**Authors:** Curtis O. Asante, Anthony H. Dickenson

**Affiliations:** Department of Neuroscience Physiology and Pharmacology, University College London, Gower Street, London WC1E 6BT, UK

**Keywords:** Rapamycin, Pain, Neuronal, Serotonergic, Spinal, Descending

## Abstract

We have recently reported the importance of spinal rapamycin-sensitive pathways in maintaining persistent pain-like states. A descending facilitatory drive mediated through spinal 5-HT3 receptors (5-HT3Rs) originating from superficial dorsal horn NK1-expressing neurons and that relays through the parabrachial nucleus and the rostroventral medial medulla to act on deep dorsal horn neurons is known be important in maintaining these pain-like states. To determine if spinal rapamycin-sensitive pathways are activated by a descending serotonergic drive, we investigated the effects of spinally administered rapamycin on responses of deep dorsal horn neurons that had been pre-treated with the selective 5-HT3R antagonist ondansetron. We also investigated the effects of spinally administered cell cycle inhibitor (CCI)-779 (a rapamycin ester analogue) on deep dorsal horn neurons from rats with carrageenan-induced inflammation of the hind paw. Unlike some other models of persistent pain, this model does not involve an altered 5-HT3R-mediated descending serotonergic drive. We found that the inhibitory effects of rapamycin were significantly reduced for neuronal responses to mechanical and thermal stimuli when the spinal cord was pre-treated with ondansetron. Furthermore, CCI-779 was found to be ineffective in attenuating spinal neuronal responses to peripheral stimuli in carrageenan-treated rats. Therefore, we conclude that 5-HT3R-mediated descending facilitation is one requirement for activation of rapamycin-sensitive pathways that contribute to persistent pain-like states.

Several studies in pain research have focused on the role of spinal 5-HT3 receptors (5-HT3Rs), which unlike all other subtypes of 5-HTRs, is the only known subtype comprising a ligand-gated ion channel [3,15]. Spinal 5-HT3Rs are located on the terminals of glutamate-releasing myelinated primary afferent fibers as well as excitatory interneurons and some NK1 projection neurons in lamina I/III [6]. In the formalin test, pre-treatment with a single intrathecal (i.t.) dose of the selective 5-HT3R antagonist ondansetron administered directly to the exposed spinal cord has been shown to attenuate neuronal hyperexcitability predominately in the second phase of the test, highlighting the peripheral and central effects of 5-HT3R activation in neuronal hyperexcitability in pain-like states [8,21]. In accordance with these studies is a study showing that ondansetron attenuates second phase formalin-induced behavioral hypersensitivity when administered 15 min prior to formalin injection [24]. Also, in rats with spinal nerve ligation (SNL, a model of neuropathy), a low dose of ondansetron was found to be effective in attenuating responses evoked by mechanical stimuli, yet was ineffective on measures of neuronal excitability in sham rats, [22]. Furthermore, in a model of osteoarthritic pain, ondansetron administered i.t. has been shown to be effective at inhibiting responses evoked by innocuous mechanical stimuli [18].

Substantiating the role of 5-HT3Rs in pain maintenance further, KO mice lacking the A subunit of the 5-HT3R, which is required for functionality of the receptor, have been shown to display normal acute pain-like responses, but attenuated ongoing hypersensitivity produced by formalin-induced inflammation [28]. Taken together, these results show that 5-HT facilitates persistent pain-like states via activation of 5-HT3Rs most likely due to an increased descending serotonergic drive from higher centres in the brain and in particular, the rostral ventromedial medulla (RVM) [23]. In accordance with these findings is a small randomized double-blind study showing that a single intravenous bolus of ondansetron alleviates the overall pain experienced by neuropathic pain patients [14].

Interestingly, not all persistent pain models involve altered descending serotonergic activity at spinal 5-HT3Rs. Carrageenan-induced inflammation has been shown to produce mechanical and behavioral hypersensitivity as well as significant neuronal plasticity [12,20]. However, electrophysiological approaches have shown that when ondansetron is administered i.t. to rats with carrageenan-induced inflammation, stimulus-evoked neuronal responses are inhibited to the same degree in both naive and carrageenan-injected rats [19]. Therefore, in this model, spinal plasticity and behavioral hypersensitivity do not require descending serotonergic activity at spinal 5-HT3Rs.

Our previous results [1,2] clearly demonstrate a link between persistent pain-like states and rapamycin-sensitive pathways at the level of the spinal cord, an area that as well as peripheral rapamycin-sensitive pathways, has gained much interest in recent times [16]. Since the persistent pain-like states we previously investigated are known to involve a descending facilitatory serotonergic drive that acts at spinal 5-HT3Rs, the aim of the current study was to investigate a possible link between spinal 5-HT3Rs and rapamycin-sensitive pathways using in vivo electrophysiological techniques.

In vivo electrophysiology studies were carried out according to a well-established protocol [25]. All studies were carried out in accordance with the UK Animals (Scientific Procedures) Act, 1986. Rats were initially anesthetized in an induction box with 4% isoflurane in a mixture of nitrous oxide (66%, v/v) and oxygen (33%, v/v). Once the rats had lost consciousness and were completely areflexic, the trachea was exposed and isolated and a cannula was inserted into the trachea and fastened with 3-0 silk threads. This was used to maintain anesthesia throughout the recording period. At this stage, the isoflurane was reduced to 2.5% (v/v) (areflexia was maintained). Rats were then secured in a stereotaxic frame and a rectal probe attached to a heating blanket was used to maintain a core temperature of 37 °C.

An incision was made through the skin along the length of vertebrae and the skin was then separated from the underlying muscle. Muscle, connective tissue and vertebrae were specifically removed from lumbar vertebral segments to expose lumbar segments L4–L5 of the spinal cord. Muscle and connective tissue immediately surrounding L4–L5 was however, kept intact, creating a well on top of the exposed spinal cord into which, drug solutions could be added. Clamps were used to stabilize and straighten the cord. The dura mater was also removed to aid drug penetration. When the set up was complete, the isoflurane was reduced to 1.8% (v/v), a level sufficient for anesthesia, whilst maintaining areflexia.

Extracellular neuronal recordings were obtained with an AC recording system (NeuroLog system, Digitimer). An electrode (parylene insulated tungsten microelectrode, 125 μm diameter, 2 MΩ, A-M systems Inc.) inserted into a head stage attached to a 3-axis manipulator was manually lowered into the exposed cord (L4–L5) to a depth of 500–1000 μM. This is an area occupied by wide dynamic range (WDR) neurons that are important in pain processing. An oscilloscope was used to isolate single neurons and a number of stimuli were applied to the receptive field.

Electrical stimuli were delivered by inserting two stimulating electrodes intradermally into the most sensitive part of the receptive field. Firstly, Aβ- and C-fiber thresholds were determined depending on their latencies to respond to stimuli (Aβ-fibers ≤ 20 ms post-stimulus; C-fibers = 90–300 ms post-stimulus). The stimulator was then set to three times C-fiber threshold and a train of 16 stimuli (0.5 Hz, 2 ms pulse width) was delivered to the receptive field to determine the number of action potentials attributable to Aβ-fibers (0–20 ms); Aδ-fibers (20–90 ms); C-fibers (90–300 ms) and post-discharge (300–800 ms) which is attributable to the repeated stimuli of nociceptive C-fibers. Mechanical stimuli (von Frey filaments ranging from 1 to 60 g force) were applied to the most sensitive part of the receptive field for 10 s. This was also the case with thermal stimuli, where increasing heat (ranging from 35 to 50 °C) was applied using a jet of water from a 60 ml syringe attached to a needle.

When determining the effect of drug or vehicle on baseline neuronal responses, only stable cells where 3 consecutive stimulus-evoked responses were within 10% of the previous result for the same test were selected for further pharmacological study, i.e., a minimum of 3 control tests were carried out prior to saline or drug administration. A ‘test’ comprising electrical, mechanical and thermal stimuli was carried out every 20 min. Saline or drug was usually administered 20 min prior to the first non-control test apart from the ondansetron pre-treatment study. In this case, ondansetron (Zofran™, Glaxo-Wellcome) or saline was administered to the spinal cord 10 min prior to rapamycin administration. Maximum changes in neuronal activity (positive or negative, raw values) from control were used for data analysis.

All drugs were administered via the i.t. route to the exposed spinal cord in a volume of 50 μl. In the studies using ondansetron to study the interactions between 5-HT3Rs and rapamycin-sensitive pathways, the spinal cord was first pre-treated with high dose ondansetron (100 μg in 50 μl saline) or saline (50 μl) 10 min prior to rapamycin (sirolimus, LC laboratories, 250 nM or 11.43 ng in 50 μl saline/DMSO, total DMSO concentration of 25%) which was also on the cord for 10 min prior to the first set of non-control tests. In separate experiments, we administered the rapamycin analogue CCI-779, which shows improved water solubility (250 nM or 12.88 ng in 50 μl saline) or a low dose of ondansetron (10 μg in 50 μl saline) to the exposed spinal cords of rats with carrageenan-induced inflammation (100 μl 2% w/v injected into the hind paw) for 20 min prior to the first set of non-control tests.

Student's *t*-tests were used to compare differences in Aβ-, Aδ- and C-fiber firing and post-discharge. Two-way ANOVA with repeated measures and Bonferroni post-tests were used to determine significance between groups for natural graded stimuli, i.e., graded mechanical and thermal stimuli.

In our first set of experiments, we studied the effects of rapamycin on neuronal responses after saline and ondansteron pre-treatment. We used rapamycin rather than the ester analogue since we had previously shown in naive animals, that this compound significantly inhibited measures of neuronal excitability including graded mechanical and thermal stimuli [1]. We also used a dose of 100 μg of ondansetron since earlier studies had shown that half this dose (50 μg) significantly inhibited mechanically- and thermally-evoked neuronal responses from naive animals [19]. We reasoned that since these neuronal responses are mediated at least in part by activity at 5-HT3Rs, the response profile produced by the administration of rapamycin in the presence of physiological saline should change when rapamycin is administered in the presence of ondansetron.

Following their physiological characterization, deep dorsal horn neurons (7 and 6 in saline and ondansetron pre-treatment groups, respectively) were randomly chosen for pharmacological investigations. Pre-drug characterizations revealed no significant differences between the neurons selected for each treatment (Table 1). We found that the inhibitory effects of rapamycin for graded mechanically- and thermally-evoked neuronal responses were significantly reduced when the spinal cord was pre-treated with the selective 5-HT3R antagonist ondansetron compared to pre-treatment with saline (Fig. 1A and B). In other words, the response profile produced by rapamycin in the presence of saline changed in the presence of ondansetron. These results imply that descending serotonergic facilitation acting at 5-HT3Rs engage rapamycin-sensitive pathways and also that baseline descending facilitatory action of serotonergic pathways at 5-HT3Rs are permissive for the inhibitory action of rapamycin. We are confident that this represents an interaction between 5-HT3Rs and rapamycin-sensitive pathways rather than the vehicle into which rapamycin is dissolved since we have previously shown that compared to vehicle, rapamycin significantly inhibits stimulus-evoked neuronal responses as well as formalin-induced neuronal hyperexcitability [1].

In our second set of experiments, the permissive action by descending serotonergic activity at 5-HT3Rs for the inhibitory action of rapamycin was further investigated in a persistent pain-like state where descending serotonergic activity at 5-HT3Rs is unaltered (at least on our neuronal measures) i.e., carrageenan-induced inflammation. We used CCI-779 rather than rapamycin since we had already shown in our most recent study that in animals which had undergone SNL to produce a neuropathic pain-like state, this compound significantly inhibits measures of neuronal excitability including graded mechanically- and thermally-evoked neuronal responses [2]. We reasoned that since carrageenan-induced inflammation is not largely dependent on activity at 5-HT3Rs whereas the inhibitory effects of rapamycin are, then the pre-drug response profile of neurons from the carrageenan-induced inflammation animals should be similar to the response profile produced after administration of the rapamycin analogue ester CCI-779.

Although the carrageenan-induced inflammation model has been shown to present behavioral hypersensitivity at 3 h post-carrageenan injection [10] that is partly due to an increase in spinal inflammatory mediators such as prostaglandins [27], in vivo electrophysiology has revealed that in this pain-like state at this specific time point, descending serotonergic activity at 5-HT3Rs is unaltered [19]. In our experiments, CCI-779 was ineffective in attenuating stimulus-evoked neuronal responses to mechanical (Fig. 2A) and thermal stimuli (Fig. 2B) at 3 h post-carrageenan administration. Likewise, low dose ondansetron (10 μg) was also ineffective in attenuating neuronal responses to mechanical (Fig. 3A) and thermal stimuli (Fig. 3B). For these experiments, we used a lower dose of ondansetron (10 μg) compared to our first set of experiments (100 μg) because this low dose of ondansetron is known to have inhibitory effects on stimulus-evoked neuronal responses from rats with SNL [22] compared to sham rats, indicating increased activity by descending facilitation acting at spinal 5-HT3Rs [2]. Taken together, these data therefore confirm that a descending serotonergic drive acting at spinal 5-HT3Rs increases in magnitude in rats with SNL but not in rats with carrageenan-induced inflammation. Interestingly, in the same model a very recent study reveals a role of rapamycin-sensitive pathways in behavioral threshold responses after injection of carrageenan into the hind paw [9]. It is therefore possible that in this model, the decreased threshold responses observed in fully awake animals are regulated by rapamycin-sensitive pathways whereas the suprathreshold neuronal responses that we obtain from our in vivo electrophysiology set up in anaesthetized animals, are not.

In a previous study [1] we showed that rapamycin produces inhibitory effects on C-fiber activity, mechanically- and thermally-evoked responses in naive animals. We also showed that spinally administered rapamycin attenuates formalin-induced hyperexcitability, a finding which was first shown by Price et al. [17]. In a separate, more recent study, we did not observe inhibitory effects by CCI-779 in control animals [2]. In our present study, CCI-779 has no effect on neuronal responses from animals with carrageenan-induced inflammation. Given that CCI-779 is an analogue of rapamycin, we would expect to see similar effects of CCI-779 on neuronal responses in animals with carrageenan-induced inflammation compared to the effects of rapamycin in naive animals because even if descending serotonergic activity is unaltered in this persistent pain-like state, there should still be an active descending serotonergic activity at spinal 5-HT3Rs that will activate rapamycin-sensitive pathways. However, as alluded to previously [2], although CCI-779 shows improved water solubility, subtle changes in pharmacokinetics which, are dependent upon the solution into which the compounds are diluted, may mean that the inhibitory effects are altered in non-pathological states and states where descending facilitation acting at spinal 5-HT3Rs is unaltered, such that 250 nM rapamycin is more efficacious than 250 nM CCI-779, at least in our hands. Importantly, rapamycin and CCI-779 inhibit neuronal responses after formalin-induced inflammation [1] and spinal nerve injury [2], respectively—two states which are significantly dependent on a descending serotonergic facilitation mediated by spinal 5-HT3Rs.

Mechanistically, although 5-HT has been shown to be important in engaging rapamycin-sensitive pathways [4,5,13,26], this is the first study using these approaches to show specifically that 5-HT3Rs are important upstream modulators of rapamycin-sensitive pathways. 5-HT3Rs are located on primary unmyelinated glutamatergic afferent terminals, excitatory interneurons as well as lamina I/III projection neurons [6,28]. We have most recently shown that rapamycin-sensitive pathways are present in spinal lamina II interneurons [2]. Identification of rapamycin-sensitive pathways in the superficial dorsal horn (as well as deeper lamina) has also been shown in other studies [7,9]. These pathways have also been shown to be important in peripheral modulation of pain processing at the level of the hind paw [11]. In contrast, there is no evidence of the presence of rapamycin-sensitive pathways in the central terminals of peripheral nerves [7]. Therefore, we propose the following: At afferent terminals in the spinal cord, 5-HT3R activation drives the excitability of spinal neurons via excitatory neurotransmitter release from spinal afferent terminals. Activation of spinal neurons and in particular, excitatory lamina II interneurons and/or projection neurons in lamina I and III results in increased activation of rapamycin-sensitive pathways within these spinal neurons that enhance dorsal horn neuronal excitability via as yet undiscovered mechanisms and these pathways are more prominent in persistent pain-like states.

Taken together, these results confirm that rapamycin-sensitive pathways are dependant at least in part upon descending serotonergic facilitation mediated by 5-HT3Rs. It is important to note that rapamycin-sensitive pathways could in theory, be activated by descending modulation at other excitatory or inhibitory receptors. However, persistent pain-like states involve shifts in neuronal thresholds and neuronal excitability to comparatively more excitatory states. Given what is already known about the importance of 5-HT3Rs in this process, excitatory 5-HT3R activation at the spinal level appears to be an important prerequisite for the activation of rapamycin-sensitive pathways.

## Figures and Tables

**Fig. 1:**
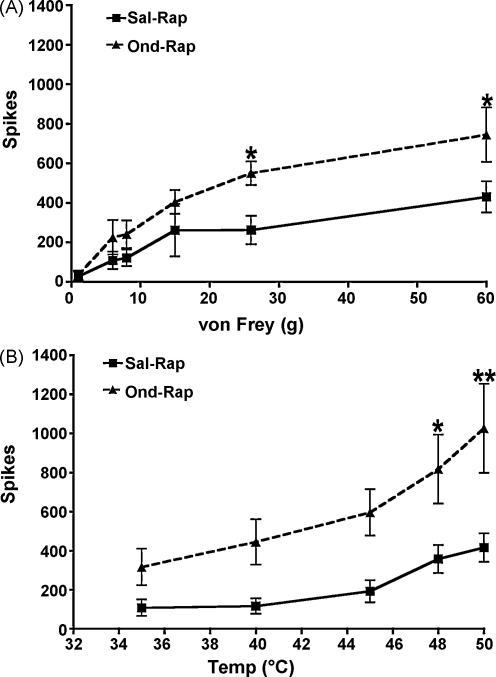
Pre-treatment of the spinal cord with 100 μg ondansetron 10 min prior to 11.43 ng rapamycin (Ond–Rap, *n* = 6) resulted in significantly less inhibition of noxious mechanically-(A) and thermally-evoked (B) responses compared to saline pre-treatment (Sal–Rap, *n* = 7, *P < 0.05, **P < 0.01).

**Fig. 2:**
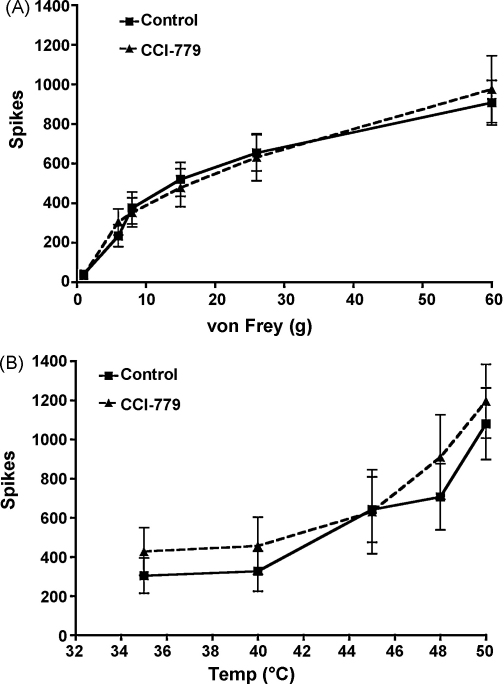
There were no effects from the administration of 12.88 ng CCI-779 (*n* = 10) to the spinal cord after 3 h establishment of carrageenan-induced inflammation (control).

**Fig. 3:**
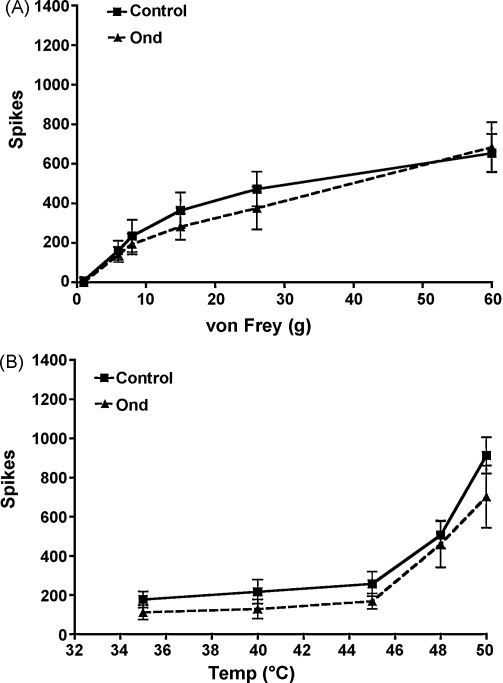
There were no effects from the administration of 10 μg ondansetron (*n* = 9) to the spinal cord after 3 h establishment of carrageenan-induced inflammation (control).

**Table 1:** Characterization of WDR neurons selected for ondansetron or saline pre-treatment prior to rapamycin.

	Saline (*n* = 7)	Ondansetron (*n* = 6)
Depth (μM)	620 ± 30	636 ± 27
Aß-fiber threshold (mA)	0.87 ± 0.06	0.66 ± 0.11
C-fiber threshold (mA)	1.60 ± 0.18	1.23 ± 0.19
Aß-fiber spikes	102 ± 25	154 ± 10
Aδ-fiber spikes	55 ± 15	70 ± 20
C-fiber spikes	235 ± 28	215 ± 38
Post-discharge spikes	140 ± 26	194 ± 64
1 g spikes	46 ± 24	27 ± 18
6 g spikes	256 ± 74	210 ± 48
8 g spikes	360 ± 115	314 ± 49
15 g spikes	457 ± 125	506 ± 75
26 g spikes	593 ± 142	669 ± 70
60 g spikes	707 ± 151	945 ± 95
35 °C spikes	248 ± 79	333 ± 93
40 °C spikes	313 ± 103	430 ± 94
45 °C spikes	547 ± 141	802 ± 95
48 °C spikes	724 ± 144	987 ± 122
50 °C spikes	858 ± 128	1120 ± 133

Data are presented as mean ± S.E.M. All responses were comparable in the two groups.

## References

[1] Asante C.O., Wallace V.C., Dickenson A.H. (2009). Formalin-induced behavioural hypersensitivity and neuronal hyperexcitability are mediated by rapid protein synthesis at the spinal level. Mol. Pain.

[2] Asante C.O., Wallace V.C., Dickenson A.H. (2010). Mammalian target of rapamycin signaling in the spinal cord is required for neuronal plasticity and behavioral hypersensitivity associated with neuropathy in the rat. J. Pain.

[3] Barnes N.M., Sharp T. (1999). A review of central 5-HT receptors and their function. Neuropharmacology.

[4] Carroll M., Warren O., Fan X., Sossin W.S. (2004). 5-HT stimulates eEF2 dephosphorylation in a rapamycin-sensitive manner in *Aplysia neurites*. J. Neurochem..

[5] Casadio A., Martin K.C., Giustetto M., Zhu H., Chen M., Bartsch D., Bailey C.H., Kandel E.R. (1999). A transient, neuron-wide form of CREB-mediated long-term facilitation can be stabilized at specific synapses by local protein synthesis. Cell.

[6] Conte D., Legg E.D., McCourt A.C., Silajdzic E., Nagy G.G., Maxwell D.J. (2005). Transmitter content, origins and connections of axons in the spinal cord that possess the serotonin (5-hydroxytryptamine) 3 receptor. Neuroscience.

[7] Geranton S.M., Jimenez-Diaz L., Torsney C., Tochiki K.K., Stuart S.A., Leith J.L., Lumb B.M., Hunt S.P. (2009). A rapamycin-sensitive signaling pathway is essential for the full expression of persistent pain states. J. Neurosci..

[8] Green G.M., Scarth J., Dickenson A. (2000). An excitatory role for 5-HT in spinal inflammatory nociceptive transmission; state-dependent actions via dorsal horn 5-HT(3) receptors in the anaesthetized rat. Pain.

[9] Gregory E.N., Codeluppi S., Gregory J.A., Steinauer J., Svensson C.I. (2010). Mammalian target of rapamycin in spinal cord neurons mediates hypersensitivity induced by peripheral inflammation. Neuroscience.

[10] Hedo G., Laird J.M., Lopez-Garcia J.A. (1999). Time-course of spinal sensitization following carrageenan-induced inflammation in the young rat: a comparative electrophysiological and behavioural study in vitro and in vivo. Neuroscience.

[11] Jimenez-Diaz L., Geranton S.M., Passmore G.M., Leith J.L., Fisher A.S., Berliocchi L., Sivasubramaniam A.K., Sheasby A., Lumb B.M., Hunt S.P. (2008). Local translation in primary afferent fibers regulates nociception. PLoS ONE.

[12] Kayser V., Guilbaud G. (1987). Local and remote modifications of nociceptive sensitivity during carrageenin-induced inflammation in the rat. Pain.

[13] Khan A., Pepio A.M., Sossin W.S. (2001). Serotonin activates S6 kinase in a rapamycin-sensitive manner in *Aplysia synaptosomes*. J. Neurosci..

[14] McCleane G.J., Suzuki R., Dickenson A.H. (2003). Does a single intravenous injection of the 5HT3 receptor antagonist ondansetron have an analgesic effect in neuropathic pain? A double-blinded, placebo-controlled cross-over study. Anesth. Analg..

[15] Millan M.J. (2002). Descending control of pain. Prog. Neurobiol..

[16] Price T.J., Geranton S.M. (2009). Translating nociceptor sensitivity: the role of axonal protein synthesis in nociceptor physiology. Eur. J. Neurosci..

[17] Price T.J., Rashid M.H., Millecamps M., Sanoja R., Entrena J.M., Cervero F. (2007). Decreased nociceptive sensitization in mice lacking the fragile X mental retardation protein: role of mGluR1/5 and mTOR. J. Neurosci..

[18] Rahman W., Bauer C.S., Bannister K., Vonsy J.L., Dolphin A.C., Dickenson A.H. (2009). Descending serotonergic facilitation and the antinociceptive effects of pregabalin in a rat model of osteoarthritic pain. Mol. Pain.

[19] Rahman W., Suzuki R., Rygh L.J., Dickenson A.H. (2004). Descending serotonergic facilitation mediated through rat spinal 5HT3 receptors is unaltered following carrageenan inflammation. Neurosci. Lett..

[20] Stanfa L.C., Sullivan A.F., Dickenson A.H. (1992). Alterations in neuronal excitability and the potency of spinal mu, delta and kappa opioids after carrageenan-induced inflammation. Pain.

[21] Suzuki R., Morcuende S., Webber M., Hunt S.P., Dickenson A.H. (2002). Superficial NK1-expressing neurons control spinal excitability through activation of descending pathways. Nat. Neurosci..

[22] Suzuki R., Rahman W., Hunt S.P., Dickenson A.H. (2004). Descending facilitatory control of mechanically evoked responses is enhanced in deep dorsal horn neurones following peripheral nerve injury. Brain Res..

[23] Suzuki R., Rygh L.J., Dickenson A.H. (2004). Bad news from the brain: descending 5-HT pathways that control spinal pain processing. Trends Pharmacol. Sci..

[24] Svensson C.I., Tran T.K., Fitzsimmons B., Yaksh T.L., Hua X.Y. (2006). Descending serotonergic facilitation of spinal ERK activation and pain behavior. FEBS Lett..

[25] Urch C.E., Dickenson A.H. (2003). In vivo single unit extracellular recordings from spinal cord neurones of rats. Brain Res. Brain Res. Protoc..

[26] Weragoda R.M., Walters E.T. (2007). Serotonin induces memory-like, rapamycin-sensitive hyperexcitability in sensory axons of aplysia that contributes to injury responses. J. Neurophysiol..

[27] Yang L.C., Marsala M., Yaksh T.L. (1996). Characterization of time course of spinal amino acids, citrulline and PGE2 release after carrageenan/kaolin-induced knee joint inflammation: a chronic microdialysis study. Pain.

[28] Zeitz K.P., Guy N., Malmberg A.B., Dirajlal S., Martin W.J., Sun L., Bonhaus D.W., Stucky C.L., Julius D., Basbaum A.I. (2002). The 5-HT3 subtype of serotonin receptor contributes to nociceptive processing via a novel subset of myelinated and unmyelinated nociceptors. J. Neurosci..

